# The Fungal Endophyte *Serendipita williamsii* Does Not Affect Phosphorus Status But Carbon and Nitrogen Dynamics in Arbuscular Mycorrhizal Tomato Plants

**DOI:** 10.3390/jof6040233

**Published:** 2020-10-19

**Authors:** Anna M. Hallasgo, Bernhard Spangl, Siegrid Steinkellner, Karin Hage-Ahmed

**Affiliations:** 1 Department of Crop Sciences, Institute of Crop Protection, University of Natural Resources and Life Sciences, Vienna, 3430 Tulln, Austria; anna.hallasgo@boku.ac.at (A.M.H.); siegrid.steinkellner@boku.ac.at (S.S.); 2 Department of Landscape, Institute of Statistics, Spatial and Infrastructure Sciences, University of Natural Resources and Life Sciences, Vienna, 1180 Vienna, Austria; bernhard.spangl@boku.ac.at

**Keywords:** arbuscular mycorrhizal fungi, endophytes, *Serendipita*, phosphorus, nitrogen, carbon, coexistence, tomato

## Abstract

Some members of the root endophytic Serendipitaceae were observed to frequently coexist with arbuscular mycorrhizal fungi (AMF), but their interactions and potential synergistic effects in plants have not yet been well elucidated. Here, we inoculated three-week-old tomato seedlings with *Serendipita indica* or *Serendipita williamsii* alone or in combination with the arbuscular mycorrhizal fungus *Funneliformis mosseae* and cultivated the plants in a greenhouse until the late vegetative stage. Our data show that the simultaneous presence of *Serendipita* spp. did not affect root colonization by AMF, proving the feasibility of their combination for future agronomic uses. The photosynthetic performance was enhanced in AM tomato plants, although growth remained unresponsive following single or dual inoculation with *Serendipita* spp. and AMF. With regard to nutrient status under dual inoculation, AMF-induced phosphorus increases remained unaffected, but nitrogen and carbon dynamics were highly altered. Specifically, the application of *S. williamsii* to mycorrhizal tomato plants significantly enhanced nitrogen concentration in the shoots, but this effect was also compensated with a carbon cost. Our findings indicate that *S. williamsii* performs differently from *S. indica* when co-inoculated with AMF, and this suggests an unknown mechanism that needs more detailed investigation.

## 1. Introduction

The rhizosphere is a habitat for a myriad of microorganisms including bacteria and fungi, where multipartite interactions take place [1,2]. While some microorganisms can inhabit plant roots and cause detrimental effects [3], others can boost performance of the host [4]. In particular, they can help overcome biotic and abiotic stress, as well as improve growth and nutrition in plants [5,6]. These microorganisms that are also ubiquitous in the environment include arbuscular mycorrhizal fungi (AMF) and root endophytic Serendipitaceae [4,7,8].

The association between plants and AMF remains one of the most essential examples of symbiosis between living organisms [9]. As predominant soil microorganisms, AMF are able to colonize the roots of more than 80% of terrestrial plants [10]. This AM symbiosis is characterized by a bidirectional relationship: Plants receive relatively immobile nutrients from the soil through the extensive hyphal network of AMF in exchange for photosynthetically fixed carbon [10,11,12,13,14]. Apart from the nutritional benefits, AMF can also enhance growth [15,16] and alleviate disease stress in plants [17,18].

In contrast, Serendipitaceae, are a recently described group of root fungal endophytes, known for their plant growth promoting properties [19]. Unlike AMF, *Serendipita* spp. grow in synthetic media and proliferate in root tissue independent of the phosphorus levels in the soil [19,20]. Two out of four recently described members of this fungal group were originally recovered from independent pot cultures of AMF [19,21]. The most well-studied member is *Serendipita indica* (syn *Piriformospora indica),* isolated from an AM fungal spore of *Funneliformis mosseae* (syn. *Glomus mosseae*), retrieved from the rhizosphere of *Prosopis juliflora* and *Zizyphus nummularia* in Northwest India [19]. Several reports have documented the ability of *S. indica* to colonize roots and consequently enhance growth [20,22,23,24,25], as well as ameliorate biotic [26,27,28,29,30] and abiotic stress in plants [31,32,33,34]. A lesser known relative of *S. indica* is *Serendipita williamsii* (syn. *Piriformospora williamsii*), which was isolated from a fungal spore of the AM fungus *Rhizophagus fasciculatus* (syn. *Glomus fasciculatum*) associated with clover roots [21]. In recent studies, *S. williamsii* surpassed the growth promoting activity of *S. indica* in *A. thaliana* [4] and also improved growth in tomato plants [22].

In an earlier study, Williams [21] uncovered an intimate association in which he detected chlamydospores of the previously recognized multinucleate *Rhizoctonia*-like isolates (recently identified as *S. williamsii*) inhabiting the intracellular structure of an AM fungus. Several decades later, other researchers reaffirmed this finding when they observed the frequent coexistence of *Serendipita* isolates with AMF in the roots of sudangrass during a trap system experiment [4]. Interestingly, single inoculation with *S. williamsii* did not promote growth in pasture legumes and ryegrass plants, but an increase of the parameter was observed when AMF was present in the system [21]. Simultaneous inoculation with *S. indica* and AMF also resulted in growth and nutrient stimulating effects in tomato plants even under salinity stress [35]. The coexistence between endophytic and mycorrhizal fungi involves a putative intimate relationship and understanding their interactions might open a window of opportunity to develop an inoculum that exceeds the outcome of a single inoculation [4].

In our study, we aimed to further investigate the coexistence between *S. indica* or *S. williamsii* and AM fungus *F. mosseae* and to elucidate their potential synergistic effects in tomato plants, a well-known host for both fungi [35]. We hypothesized that the combination of endophytic and mycorrhizal fungi would influence AMF root colonization, growth, photosynthetic activity, and nutrient status in tomato plants.

## 2. Materials and Methods

### 2.1. Fungal Material and Inoculum Production

*Serendipita* species, including *Serendipita indica* (DSM 11827) and *S. williamsii* (DAR 29830), kindly provided by Michael Weiß (University of Tübingen, Germany), were propagated on a modified Hill and Käfer Medium [36] in the dark at 24 °C. To produce the inoculum, small plugs of four-week old cultures were transferred in 250 mL Erlenmeyer flasks containing 150 mL of liquid Hill and Käfer Medium and incubated in an orbital shaker for three weeks in the dark at 24 °C. Fungal cultures were filtered and homogenized using a high speed blender, three times, for 30 s each. Subsequently, the homogenized mycelial fragments were centrifuged at 4000 rpm for five minutes at room temperature. The supernatant was discarded, and the pellets were washed with autoclaved dH_2_O. The concentration of the mycelial fragments was determined using a Fuchs-Rosenthal hemocytometer and adjusted to 3 × 10^5^ cfu·mL^−1^ of autoclaved dH_2_O [24]. To verify the viability of the fungal propagules, inoculum suspensions of *Serendipita* spp. were plated in a solidified Hill and Käfer Medium and incubated at 24 °C.

The AMF inoculum was produced in the greenhouse using *Plantago lanceolata* L. as a host plant. Propagules of *Funneliformis mosseae* (BEG 12) were inoculated into pots containing a mixture of sand (Quarzsand 0–3 mm, Quarzwerke Österreich GmbH, Melk, Austria), expanded clay (Seramis, Seramis GmbH, Mogendorf, Germany) (1:1, *v*/*v)*, and seeds of *P. lanceolata*. The plants were grown in a greenhouse for several months, and the roots were regularly checked for AMF colonization. A mixture of the AMF colonized substrate (spores, hyphae, sand, and expanded clay) and root fragments of *P. lanceolata* was used as the AMF inoculum.

### 2.2. Plant Material, Inoculation, and Cultivation

Seeds of tomato (*Solanum lycopersicum* L. cv. “Kremser Perle”) were surface-sterilized with diluted household bleach (1.4% NaClO) for ten minutes and then rinsed three times with autoclaved dH_2_O. The surface-sterilized seeds were sown into pots filled with double autoclaved perlite (Granuperl S 3–6, Knauf Perlite GmbH, Vienna, Austria) and cultivated in a growth chamber at 24 °C with a 14 h photoperiod.

After three weeks, the seedlings were characterized by the appearance of the first true leaf and then harvested from the perlite. Next, the roots were soaked in the *Serendipita* spp. inoculum suspension (3 × 10^5^ cfu·mL^−1^) for 24 h [22]. Roots of the control plants were submerged in autoclaved dH_2_O. After 24 h, the sample roots were stained with an ink-vinegar solution [37] to confirm root colonization by *Serendipita* spp. Prior to transplanting, 10 mL of the AMF inoculum was added to the planting hole of the AMF treated plants (+AMF). Non-mycorrhizal (−AMF) pots obtained 10 mL of microbial wash to correct potential differences in their microbial communities [38]. The microbial wash was produced by wet sieving a mixture containing the AMF inoculum and dH_2_O (1:6, *v*/*v*) through a 38 µm sieve.

Seedlings were transplanted in 10 cm pots filled with soil (Aussaaterde, Gramoflor GmbH & Co. KG, Vechta, Germany), sand (Quarzsand 0–3 mm, Quarzwerke Österreich GmbH, Melk, Austria), and clay (Liapor fit 1–4 mm, Lias Österreich GmbH, Fehring, Austria) (1:1:1, *v*/*v*/*v*). The substrate had a pH of 6.56 and contained the following elemental concentrations in kg^-1^ soil: 25.86 mg P, 68.97 mg K, 123.49 µg Fe, 11.22 µg Mn, and 0.97 µg Zn. Each plant received 30 mL of standard low phosphorus nutrient solution per week starting from the second week after transplanting, with the following components L^−1^: 0.09 mm P, 3.57 mm N, 1.59 mm K, 3.07 mm Mg, 3.47 mm Ca, 4.58 mm S, 98.78 µm Fe, 4.76 µm B, 3.79 µm Mn, 2.09 µm Zn, 2.09 µm Cu, 0.49 µm Mo, and 3.79 µm Cl [39]. Irrigation with tap water was done twice per week or when needed. The experimental set-up consisted of the following treatments: (i) control, (ii) *S. indica* and (iii) *S. williamsii* all with (+AMF) and without AMF (-AMF). Each treatment featured 15 replicate pots with a total number of 90 pots. Plants were grown in the greenhouse and arranged in a randomized complete block design. Day and night temperatures were 24 and 18 °C, respectively, with relative humidity of 60%. A 16 h photoperiod was used, and additional light was provided when the outside photosynthetically active radiation (PAR) was below 367.43 µmol m^−2^·s^−1^.

### 2.3. Photosynthetic Performance

The performance of photosystem II was assessed using a portable chlorophyll fluorometer (PAM 2500, Walz, Germany). The minimal fluorescence yield (Fo), maximal fluorescence yield (Fm), and maximum photochemical quantum yield of photosystem II (Fv/Fm) were measured using the youngest fully expanded leaf of the dark adapted (30 min) tomato plants.

### 2.4. Harvest and Nutrient Analysis of Tomato Shoots

Tomato plants were harvested nine weeks after transplanting. The fresh weight and length of the shoots and roots were determined. The shoot material was oven-dried at 65 °C, and the dry weight was measured. The roots were then either stored in 50 mL centrifuge tubes filled with 30% ethanol and kept at 4 °C until staining or stored at −80 °C until DNA extraction.

Oven-dried tomato shoots were pooled into four samples per treatment. Pooled samples were milled into a fine powder using a Retsch Knife Mill Grindomix GM200 plant mill (Retsch GmbH, Haan, Germany). Thereafter, the samples were dried at 105 °C prior to elemental analysis. The macronutrients such as phosphorus (P), calcium (Ca), potassium (K), and magnesium (Mg), as well as the micronutrients such as iron (Fe), manganese (Mn), and zinc (Zn) were analyzed by inductively coupled plasma-atomic emission spectroscopy (ICP-OES) Optima 8300 DV (PerkinElmer, Waltham, MA, USA). The ground sample of shoot tissue (200 mg ± 10 mg) was subjected to acid digestion by directly adding 8 mL of HNO_3_ in an Xpress microwave vessel (CEM, Buckingham, UK) containing the sample. The next day, 2 mL of H_2_O_2_ was added to the mixture, and digestion was carried out using the microwave instrument MARS 6 (CEM, Buckingham, UK). Then, the digested solution was diluted to 50 mL using dH_2_O. The analysis was performed with ICP-OES using 426 mL of the diluted sample, 5.72 mL of dH_2_O, and 0.769 mL of the reference standard.

The carbon and nitrogen ratio (C/N) was assessed according to the Dumas combustion method [40]. Approximately 10–15 mg of the ground sample was weighed in a tin container and loaded into the Vario Macro Cube automatic sampler (Elementar, Langenselbold, Germany). The combustion tube was filled with the tin containing the sample. Eventually, the combustion started at 960 °C and accelerated to 1800 °C, thus converting carbon to CO_2_ and nitrogen to N_2_. The carbon dioxide and nitrogen were separated and then detected by a thermocouple device.

### 2.5. Detection of AMF in Colonized Roots

AMF structures were visualized using the ink-vinegar staining procedure [37]. Tomato roots were cleared by immersion in 10% potassium hydroxide (KOH) for five minutes at 90 °C. The roots were rinsed with running tap water to remove the KOH. Afterwards, a 5% ink-vinegar solution was added to the roots and boiled for 20 min at 90 °C. Finally, the stained roots were incubated in tap water with few drops of vinegar for 30 min and then stored in 30% ethanol until microscopic evaluation.

AMF root colonization was evaluated using the method of McGonigle et al. [41]. The stained roots were cut into small pieces (approximately 1 cm), and 15 random samples were mounted onto a microscope slide using acid glycerol, with the following components: 700 mL glycerol, 230 mL dH_2_0, and 70 mL 1% HCl. Two slides per sample (i.e., 30 root pieces), with 100 intersections per slide, were used for the evaluation. To observe the intraradical AMF structures (hyphae, arbuscules, and vesicles), the slide was placed under a light microscope (Olympus BX53) and analyzed using 200 × magnification. To calculate the relative amount of different AMF structures, the following formulae were used:

(1) $$\%\;\mathrm{AC}=\;\frac{\mathrm{number}\;\mathrm{of}\;\mathrm{intersections}\;\mathrm{with}\;\mathrm{the}\;\mathrm{presence}\;\mathrm{of}\;\mathrm{arbuscules}}{\mathrm{total}\;\mathrm{number}\;\mathrm{of}\;\mathrm{intersections}\;\mathrm{examined}\times 100}$$

(2) $$\%\;\mathrm{VC}=\;\frac{\mathrm{number}\;\mathrm{of}\;\mathrm{intersections}\;\mathrm{with}\;\mathrm{the}\;\mathrm{presence}\;\mathrm{of}\;\mathrm{vesicles}}{\mathrm{total}\;\mathrm{number}\;\mathrm{of}\;\mathrm{intersections}\;\mathrm{examined}\times 100}$$

(3) $$\%\mathrm{TC}=\;\frac{\mathrm{total}\;\mathrm{number}\;\mathrm{of}\;\mathrm{intersections}\;\mathrm{examined}-\mathrm{negative}\;\mathrm{intersect}}{\mathrm{total}\;\mathrm{number}\;\mathrm{of}\;\mathrm{intersections}\;\mathrm{examined}\times 100}$$

### 2.6. DNA Extraction and Detection of Serendipita spp. in Colonized Roots by Nested PCR

The total DNA of the tomato roots (4 samples per treatment) was extracted from a ≤ 100 mg sample using a DNEasy Plant Mini Kit (Qiagen, Hilden, Germany) according to the manufacturer’s instructions. In the first polymerase chain reaction (PCR), 852 bp and 1.1 kb fragments of the translation elongation factor (TEF) 1-α gene of *S. indica* and *S. williamsii*, respectively, were amplified. The primers SiTEF2879f (5′-GTTTCTTTGTCGTCTCGTTC-3′) and SiTEF3711r (5′-GAAAATGTGGTGGGTTTACG-3′) for the *S. indica* TEF fragment were designed using the Primer-BLAST algorithm [42] on the TEF 1-α gene (AJ249911.2). For amplification of the fragment of the TEF region of *S. williamsii*, the primers EF1-0728f [43] and EF1-1620r [44] were used. The total reaction volume in each tube was 25 µL and included 18.8 µL ddH_2_0, 2.5 µL reaction buffer (10×), 0.5 µL dNTPs, 0.5 µL for each primer, 0.2 µL Taq polymerase (Taq DNA Polymerase, VWR, Vienna, Austria), and 2 µL DNA template. Amplification was performed in an Eppendorf Nexus GX2 thermocycler (Eppendorf Austria GmbH, Vienna, Austria) with the following cycling parameters: 95 °C for 2 min, 20 cycles at 95 °C for 20 s, 58 °C (Si)/54 °C (Sw) for 30 s, and 72 °C for 50 s, with a final step at 72 °C for 5 min. In a second PCR run, smaller areas of the previously amplified TEF fragments were targeted. *S. indica* specific primer pairs (SiTEF3210f 5′-GTGTGTGGAGAGCTACAACGA-3′) and SiTEF3210r 5′-CGCGCTCTTCGTAACTGGAA-3′) were designed using the Primer-BLAST algorithm [42] on the TEF region (AJ249911.2). For *S. williamsii*, the portion of the TEF region amplified by the primers TEF420f/TEF420r [45] was sequenced (LGC Genomics GmbH, Berlin, Germany), and thereafter the reverse primer SwTEF1r (5′-GATACAACGCGGGGGAGTTC-3′) was designed using the Primer-BLAST algorithm [42]. The primer TEF420f [45] was used as the forward primer for *S. williamsii*. The reaction volumes and compounds were used as previously described, apart from adding 2 µL of the diluted PCR product (1:100) from the first PCR. The cycling parameters were similar to those of the first PCR apart from the annealing temperature of 60 °C and the use of 30 cycles. The presence of amplified PCR products was confirmed using a 2% agarose gel in a 1× TAE buffer.

### 2.7. Statistical Analysis

A two-way ANOVA with the fixed factors *Serendipita* and AMF was performed in the software IBM SPSS Statistics (ver. 26, IBM Incorporation, Armonk, NY, USA). The factor *Serendipita* had three levels including control, *S. indica* and *S. williamsii* while the factor AMF had two levels including − AMF (without AMF) and + AMF (with AMF). Data were checked for homogeneity of variance using Levene’s test. In addition, a simple effect test was performed when interaction effects were found to be significant. Moreover, principal component analysis and k-means clustering of the nutrient and AMF root colonization variables were conducted using the package “factoextra” in the R software (ver. 3.6.3, R Foundation for Statistical Computing, Vienna, Austria), as performed in Kalaji et al. [46]. The PCA biplot and the cluster map were produced in R. All other graphs and figures were created using Sigma Plot (ver. 14, Systat Software Inc., San Jose, CA, USA).

## 3. Results

### 3.1. Fungal Colonization

#### 3.1.1. Root Colonization by AMF

**Figure 1:**
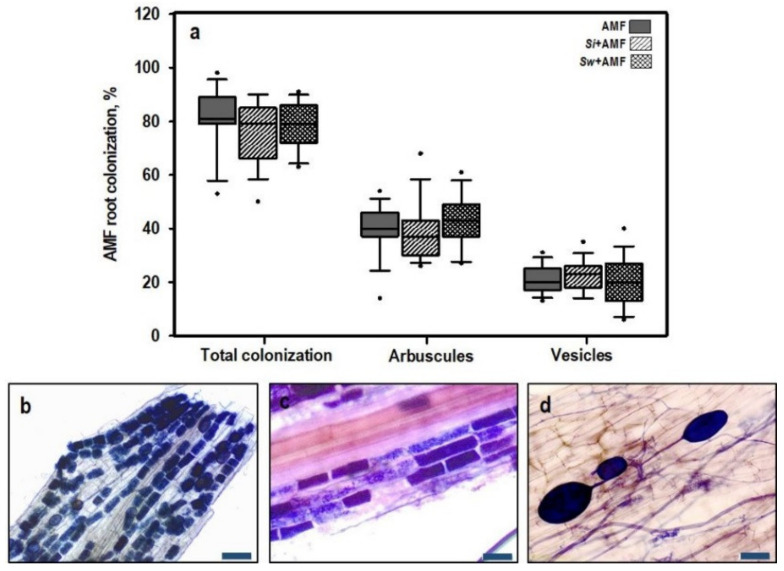
AMF root colonization in tomato roots nine weeks after transplanting including the % total root colonization, % arbuscules, and % vesicles (**a**). There were no significant differences detected between the treatments (ANOVA, *p* < 0.05, *n* = 15). Different parts showing an intensely colonized root piece (**b**), arbuscules (**c**), and vesicles (**d**) in the tomato roots. Roots were stained using the ink-vinegar method of Vierheilig et al. [37]. Scale bar: 100 µm (**b**) and 50 µm (**c**,**d**).

Three independent ANOVAs were performed for the total, arbuscular, and vesicular root colonization by AMF, and we found no significant differences between treatments for each parameter (Table 1 and Figure 1a). Tomato roots were intensely colonized by AMF (Figure 1b), with a colonization percentage of between 50 and 100%. Moreover, the arbuscular root colonization (Figure 1c) was between 15 and 70%, while the amount of vesicles (Figure 1d) was between 10 and 40%. No AMF structures could be detected in non-mycorrhizal tomato plants.

#### 3.1.2. Root Colonization by *Serendipita* spp.

**Figure 2:**
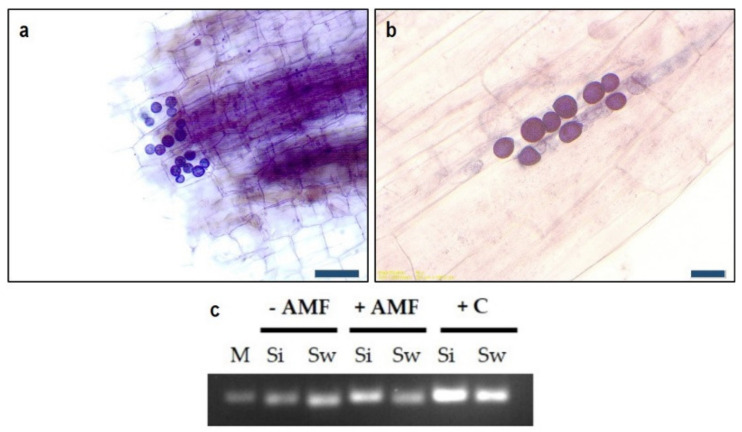
Root colonization by *Serendipita* spp. including chlamydospores of *S. indica* (**a**) and *S. williamsii* (**b**) observed in the mycorrhizal roots of tomato plants. Roots were stained using the ink-vinegar method of Vierheilig et al. [37]. Scale bar: 50 µm (**a**); 20 µm (**b**). Verification of root colonization of *S. indica* (Si, 189 bp) and *S. williamsii* (Sw, 180 bp), respectively, by nested PCR (**c**). M: marker (200 bp), +C: positive control from pure fungal cultures.

Through staining and microscopic confirmation, dense mycelial networks of *Serendipita* spp. were found on the surface of the tomato roots 24 h after inoculation. Structures of *Serendipita* spp., such as chlamydospores of *S. williamsii* (Figure 2a) and chlamydospores of *S. indica* (Figure 2b) in the mycorrhizal tomato plants nine weeks after transplanting, were also observed during the microscopic evaluation. Root colonization by *S. indica* and *S. williamsii*, respectively, was further confirmed by nested PCR (Figure 2c). *S. indica* and *S. williamsii* could be detected in the respective samples. *Serendipita* spp. were not detected in uninoculated control plants.

### 3.2. Plant Growth and Performance of Photosystem II

The shoot length and shoot dry weight in tomato plants nine weeks after transplanting were unaffected by the main factors of “*Serendipita*” and “AMF”, as well as the “*Serendipita*” × “AMF” interactions (Table 2 and Table 3).

**Figure 3:**
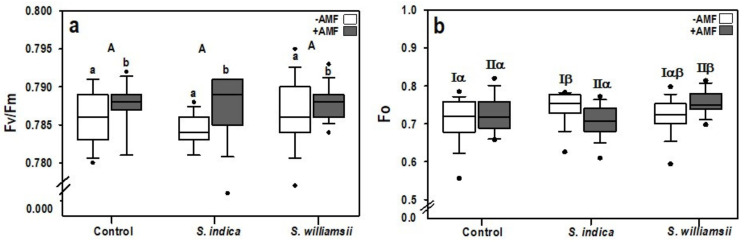
Chlorophyll fluorescence parameters including the maximum photochemical quantum yield of photosystem II (Fv/Fm) (**a**) and the minimal chlorophyll fluorescence yield (Fo) (**b**). Upper-case letters indicate differences in the main factor ‘*Serendipita*’. Lower-case letters indicate differences in the main factor ‘AMF’ (ANOVA, *p* < 0.05, *n* = 15). Greek letters were used to highlight significant interaction effects based on the simple effect analysis for −AMF(I) and +AMF(II). Columns followed by the same letters are not significantly different.

The main factor *Serendipita* did not affect the chlorophyll fluorescence parameters (Fo, Fm, and Fv/Fm) (Table 2). However, the main factor AMF had a significant influence on the maximum photochemical quantum yield of photosystem II (Fv/Fm) (*F*_(1,84)_ = 5.246, *p* = 0.025). The Fv/Fm values of AMF inoculated plants (+AMF) were significantly higher than those of non-mycorrhizal plants (−AMF) (Figure 3a). The highest level was observed when *S. indica* was co-inoculated with AMF compared to plants singly inoculated with the endophyte. In addition, a simple effect test was conducted on the minimal chlorophyll fluorescence (Fo) yield because the statistical test showed a significant interaction between *Serendipita* × AMF (*F*_(2,84)_ = 4828, *p* = 0.010) (Table 2). The significant interaction was represented by a higher Fo in *S. indica* treated plants than the control in the −AMF treatment. An increase in Fo in *S. williamsii* + AMF treated plants was also observed compared to both the control with AMF and *S. indica* + AMF inoculated plants (Figure 3b). A significant interaction effect between *Serendipita* and AMF was found in the maximum chlorophyll fluorescence (Fm), but the simple effect test yielded no significant differences between the different levels of the main fixed factors.

### 3.3. Nutrient Status in Tomato Shoot

#### 3.3.1. Macronutrients

**Figure 4:**
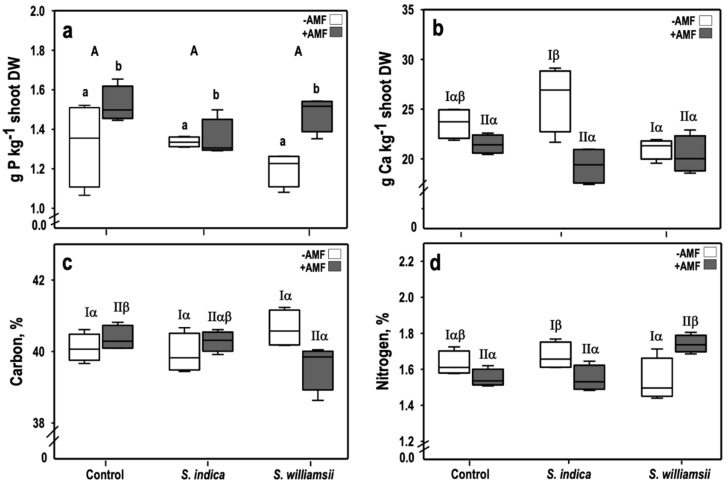
Macronutrient concentration in tomato shoots nine weeks after transplanting: phosphorus (**a**) and calcium (**b**) (in g·kg^−1^ shoot dry weight) and carbon (**c**) and nitrogen (**d**) (in %). Upper-case letters indicate differences in the main factor *‘Serendipita’*. Lower-case letters indicate differences in the main factor ‘AMF’ (ANOVA, *p* < 0.05, *n* = 4). Greek letters were used to highlight significant interaction effects based on the simple effect analysis for −AMF(I) and +AMF(II). Columns followed by the same letters are not significantly different.

Phosphorus concentration was significantly affected by the factor AMF (*F*_(1,18)_ = 12.412, *p* = 0.002) (Table 4). This influence was characterized by a 13% increase in P concentration in the +AMF compared to the −AMF plants (Figure 4a). The factors *Serendipita* and *Serendipita* × AMF did not affect the P status in tomato plants.

Although the factor, AMF, significantly affected the calcium concentration in tomato shoots (*F*_(1,18)_ = 16.819, *p* = 0.001), this effect was not exclusive since the *Serendipita* × AMF interaction was also observed to be significant (*F*_(2,18)_ = 20.988, *p* = 0.012) (Table 4). This interaction effect manifested as a 20% increase in Ca concentration in *S. indica* compared to *S. williamsii* but not compared to the control uninoculated plants in the −AMF treatment (Figure 4b).

Both carbon and nitrogen, were unaffected by the main factors AMF and *Serendipita* (Table 4). Nevertheless, these two elements were influenced by the *Serendipita* x AMF interactions (*F*_(2,18)_ = 5.414, *p* = 0.014) and (*F*_(2,18)_ = 11.008, *p* = 0.001), respectively. For carbon, a significant interaction was found in the +AMF treatments (Figure 4c). Specifically, a reduction was observed between the control plants (40.37 ± 0.35%) and the *S. williamsii* + AMF inoculated plants (39.60% ± 0.65). For nitrogen, a significant interaction occurred in both the −AMF and +AMF treatments. In the −AMF treatment, a significant interaction was noticeable as an increase of N in *S. indica* (1.67 ± 0.08%) compared to *S. williamsii* (1.54 ± 0.12%) inoculated plants (Figure 4d) but not in comparison to the control untreated plants. In the +AMF treatment, co-inoculation with *S. williamsii* + AMF resulted in an increase in N (1.74 ± 0.05%) as compared to both the control (1.55 ± 0.05%) and *S. indica* + AMF (1.55 ± 0.07%) plants.

**Figure 5:**
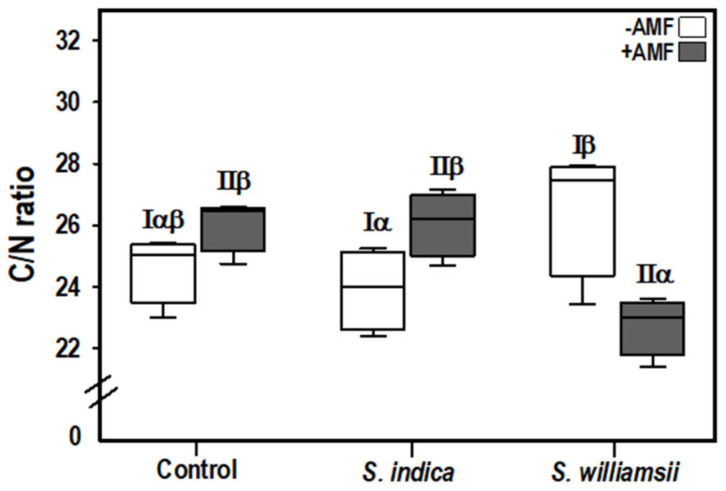
Carbon to nitrogen ratio (C/N) in the tomato shoots nine weeks after transplanting. (ANOVA, *p* < 0.05, *n* = 4). Greek letters were used to highlight significant interaction effects based on the simple effect analysis for −AMF(I) and +AMF(II).

The CN ratio was significantly influenced by the *Serendipita* × AMF interaction (*F*_(2,18)_ = 12.369, *p* = 0.000) (Table 4). This interaction was apparent between the +AMF plants (Figure 5). Specifically, the CN ratio was reduced by 13% when *S. williamsii* was combined with AMF compared to the control mycorrhizal plants.

#### 3.3.2. Micronutrients

**Figure 6:**
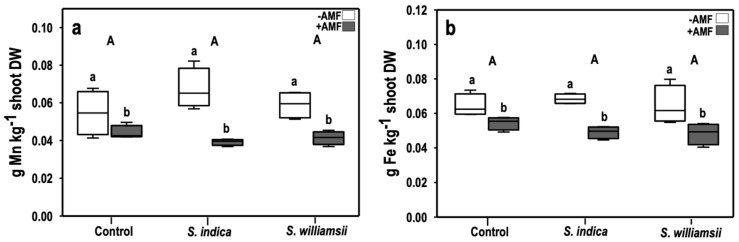
Manganese (**a**) and iron (**b**) concentrations (in g·kg^−1^ shoot dry weight) in tomato shoots nine weeks after transplanting. Upper-case letters indicate the differences in the main factor ‘*Serendipita*’. Lower-case letters indicate the differences in the main factor ‘AMF’. Columns followed by the same letters are not significantly different (ANOVA, *p* < 0.05, *n* = 4).

The manganese (Mn) concentration in tomato shoots was significantly affected by the main factor AMF (*F*_(1,18)_ = 37.199, *p* = 0.000) (Table 5). This manifested as a 33% reduction of Mn in the +AMF compared to the −AMF treated plants (Figure 6a).

The iron concentration in the tomato shoots was also considerably affected by the main factor AMF (*F*_(1,18)_ = 34.548, *p* = 0.000) (Table 5). This was characterized as a 22% reduction of Fe in the +AMF compared to the −AMF treated plants (Figure 6b). The zinc concentrations in the tomato shoots were similar under all treatments (Table 5).

### 3.4. Correlation between Nutrients and AMF Root Colonization

**Figure 7:**
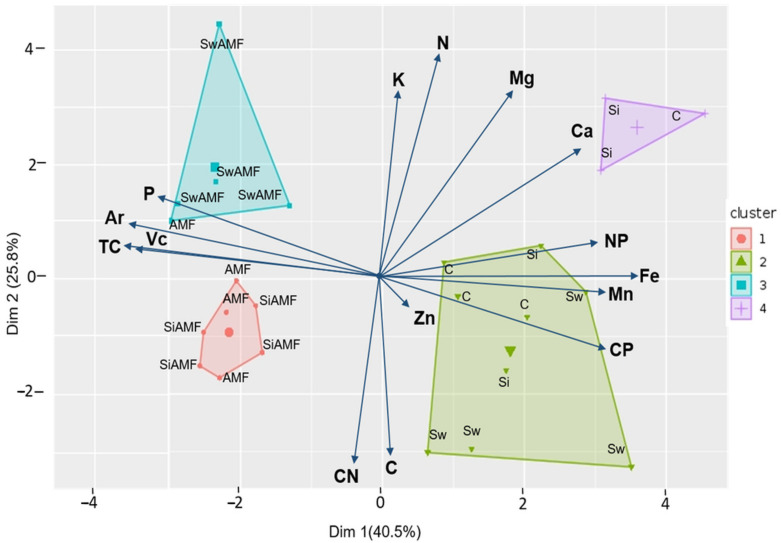
Principal component analysis of tomato samples with regards to macro and micronutrient concentrations in tomato shoots, as well as AMF colonization in the roots. The first two axes represent 40.5 and 25.8% variation in the data. Nutrient variables include potassium (K), nitrogen (N), magnesium (Mg), calcium (Ca), nitrogen to phosphorus ratio (NP), iron (Fe), manganese (Mn), carbon to phosphorus ratio (CP), zinc (Zn), carbon (C), carbon to nitrogen ratio (CN), and phosphorus (P). The AMF root colonization parameters include total colonization (TC), arbuscular colonization (Ar), and vesicular colonization (Vc). Data points represent the treatments including the control (C), *Si* (*S. indica*), *Sw* (*S. williamsii*), and arbuscular mycorrhizal fungi (AMF).

Among all analyzed nutrients, only phosphorus was drawn towards the same axis with AM*_F_* root colonization while the other nutrients, particularly manganese and iron, were negatively correlated (Figure 7). In addition, all samples treated with AMF were correlated with phosphorus and AMF root colonization parameters (e.g., total colonization (TC), arbuscular colonization (Ar), and vesicular colonization (Vc)). *S. indica* + AMF plants shared the same cluster with AMF plants, while *S. williamsii* + AMF was found in a different group. All non-mycorrhizal treatments were located on the opposite side of the axis and were more similar in terms of other nutrients such as Ca, Mn, Fe, and Zn.

## 4. Discussion

In our study, we demonstrated that the root endophytes *Serendipita indica* and *S. williamsii* coexist with the arbuscular mycorrhizal fungus *F. mosseae* in tomato roots, as shown via microscopic and molecular analyses. The simultaneous presence of either *S. indica* or *S. williamsii* did not affect the AMF colonization parameters in tomato plants. This outcome might be due to the different colonization strategies and niche preferences among the endophytic and mycorrhizal fungi. It was reported that *S. indica* can develop even in dead host cells [47], while AMF can only proliferate in living host tissue [10]. These characteristics indicate less competition for space and resources, giving each other a venue to coexist. The association between *Serendipita* and AMF was previously emphasized in two independent studies. In an earlier study, Williams [21] detected chlamydospores of *S. williamsii* in the intracellular structure of an AM fungus and he found positive growth effects when AMF was added to the plant-endophyte system. Several decades later, Venneman et al. [4] observed the frequent coexistence between *Serendipita* isolates and AMF in the roots of sudangrass but they did not investigate the potential effects of the combined inoculation in plants. In our research, the introduction of *S. williamsii* to AM tomato plants did not affect AMF root colonization, indicating that it may occupy a niche in the root similar to *S. indica*. The absence of adverse effects on AMF colonization upon inoculation with *Serendipita* spp. suggests the possibility of coexistence, proving the feasibility of their combination for future agronomic uses. However, variable amounts of *S. indica* inoculum and different inoculation methods caused a decline in AMF root colonization in tomato [35], but an increase of this parameter in mycorrhizal sweet basil plants [33]. This indicates that, to a certain extent, the amount of inoculum, the method of inoculation, the stage of colonization, and the plant and mycorrhizal species dictate the function of the tripartite interaction, but this relationship requires further confirmation.

Growth in tomato plants was unaffected following single or dual inoculation with *Serendipita* spp. and AMF. Even though we used a low nutrient regime in our study, we did not observe a negative growth response, especially as both mycorrhizal and endophytic fungi can compete with plants for nutrients and other resources [14,48,49,50]. Contrary to our results, a combination of *S. indica* or *S. williamsii* and AM fungus *R. irregularis* increased growth in sweet basil [33] and pasture legumes [21], respectively. Several factors, such as the variabilities in the experimental set-up, environmental conditions, and plant species likely contributed to these disparities, as has been shown in other studies [22,24]. Nevertheless, the AM tomato plants showed higher photosynthetic activity than their non-mycorrhizal counterparts. AM fungi play a role in improving the function of photosystem II through stabilizing electron transport at both the receptor and donor sides [51]. The higher photosynthetic activity in the AM plants did not reflect growth enhancement because a substantial amount of photosynthates was also supplied to the fungal symbiont to maintain their mycorrhizal networks and complete their life cycles [11,14].

The addition of AMF considerably increased the phosphorus (P) concentration in the AM tomato plants compared to their non-mycorrhizal counterparts. Positive effects of AMF inoculation on P status in tomato plants was also shown in the study of Hart et al. [52]. Phosphorus is an essential nutrient required for various biological and chemical processes in plants [53]. However, it is poorly available in the soil [10], which poses a challenge to crop production systems. The use of biological agents such as AMF is among the most well-recognized measures to improve P bioavailability [14]. Mycorrhizal hyphae can penetrate the soil more efficiently than plant roots [54,55], thus making AMF an undisputable fungal partner in enhancing nutritional status in plants. In contrast to AMF, the role of *Serendipita* spp. in plant P nutrition is still controversial [56]. For instance, some authors reported the absence of P stimulating factor of *S. indica* in tobacco [25], barley [23], chickpea, [57] and potato [58], similar to our findings. Due to the inability of this endophyte to affect P status in plants, several studies were focused on the combinatory effects of *S. indica* and potential phosphate solubilizing bacteria (PSB) [56,59]. Interestingly, *S. indica* was found to host an endobacterium called *Rhizobium radiobacter* [60], whose role in the tripartite interaction is not yet fully understood [61]. Nevertheless, other authors showed a positive outcome on P uptake after inoculating plants with *S. indica* [62,63,64], the underlying mechanism of which could include the lowering of pH through the production of organic acids [65], stimulating acid phosphatase activity [63], and inducing high affinity phosphate transporter genes, especially under phosphate deprived conditions [64]. It is not known whether *S. williamsii* can affect P status in plants, but our findings suggest that it acts similar to *S. indica;* it inhabits the roots next to the AMF without altering the phosphate stimulating function of the latter.

To the best of our knowledge, this is the first study that exhibits the synergistic effects of *Serendipita* spp. and AMF on the CN dynamics in tomato plants. Specifically, the addition of *S. williamsii* boosted the N concentration in AM tomato shoots. Soil microorganisms play a vital role in both the immobilization and mineralization of organic nitrogen [66] and the nitrification or denitrification of inorganic N [10]. Unlike phosphate, nitrogen sources such as nitrate and ammonium are relatively mobile in the soil; thus, plant roots take them up even in a non-mycorrhizal state [10]. Nevertheless, the impact of AMF in influencing N uptake has received much attention recently [67]. For example, some authors showed an increase of nitrogen in AM tomato plants due to elevated levels of amino acids [68], which are crucial for N uptake in mycorrhizal symbiosis [69]. Conversely, other authors reported negative effects of AMF in plant N acquisition, which might be due to fungal N retention [9,70,71]. AM fungi require nitrogen for their growth and maintenance, and, under a limited N supply, the fungus acts as the first N sink [72]. As a consequence, AMF provides the host plant with surplus N [73,74]. In our experimental system, we assume that the mycorrhizal fungi can serve as an N sink and that the presence of *S. williamsii* ameliorates the process such that a considerable amount of N is transferred to the plant. In addition, the increase of nitrogen in the *S. williamsii* + AMF treated plants was compensated with the carbon cost. Fellbaum et al. [75] showed that carbon availability significantly triggers nitrogen uptake and transport in AM plants. We also cannot rule out the possibility that AMF can become parasitic in this type of system because mycorrhizal fungi are considered to be a major sink for photosynthates [14,48]. Although *S. indica* and AMF coexisted, their interactions did not alter the CN dynamics in tomato plants. The work of Zucarro et al. [76] provided insights into the genome of *S. indica* and revealed the absence of genes that encode for the uptake and reduction of nitrate. Therefore, *S. williamsii* potentially performs differently from *S. indica* when co-inoculated with AMF, which points to an unknown mechanism that needs more detailed investigation.

Single inoculation with *S. indica* enhanced calcium (Ca) concentration in tomato shoot in comparison to the *S. williamsii* treated plants, although it was not significantly different from the control untreated plants. Calcium is relatively mobile in the soil, but the presence of *S. indica* might be an important strategy to enhance Ca uptake by plants especially in Ca deficient soils.

In our experiment, we observed a consistent decline in manganese and iron in mycorrhizal tomato plants. AMF has been reported to reduce the microbial communities responsible for Mn^+IV^ reducing potential, thereby decreasing Mn stimulating activity in the soil [77]. Other authors showed the ability of AMF to regulate higher concentrations of micronutrients by sequestering Mn granules [78]. Iron is poorly soluble in the soil, and a common strategy developed by fungi to efficiently take up this element involves the secretion of siderophores [79]. The effects of AMF on iron accumulation in plants may also vary depending on the inoculation method. For example, AMF mixed with the substrate resulted in greater Fe accumulation in the tomato shoots, but the element was reduced when the inoculum was added under the seedlings [80]. It was also highlighted that AMF can increase the concentrations of certain elements in the roots but decrease the transport to the shoots [10,81,82], possibly due to sequestration in the hyphae [83]. Inoculation with *S. indica* alone resulted in increased Fe concentrations in lettuce leaves under nutrient deficient soil [84], as well as in wheat under nutrient sufficient conditions [85]. Interestingly, the combination of *S. indica* and *Azotobacter chroococcum* decreased the Fe concentration in wheat plants, which can be attributed to the lesser tolerance of the bacterial partner to nutrient deficiency [85]. Moreover, we cannot exclude the fact that the presence of both endophytic and mycorrhizal fungi in the roots could also entail competition of the micronutrients with their plant partner.

## 5. Conclusions

To summarize, we confirmed the coexistence of root endophytic *Serendipita* spp. and arbuscular mycorrhizal fungus *F. mosseae* in tomato roots. Furthermore, we found a highly dynamic relationship between *S. williamsii* and AMF: This combination enhances the nitrogen concentration in tomato shoot without compromising the phosphorus stimulating function of AMF. This synergistic effect offers new insight into the use of microbial combinations to enhance nutrient acquisition in plants. However, the optimum amount of inoculum and the method of inoculation for both endophytic and mycorrhizal fungi must be investigated in to maximize the potential of their combination. Moreover, we observed that *S. williamsii* performs differently from *S. indica* when co-inoculated with AMF, indicating an unknown mechanism that needs further studies.

## Figures and Tables

**Table 1:** Results of the one-way ANOVA (*p*-values) for the arbuscular mycorrhizal fungi (AMF) root colonization parameters in tomato plants nine weeks after transplanting (*p* < 0.05, *n* = 15).

	AMF Root Colonization		
	Total Colonization	Arbuscules	Vesicles
Treatment*s*	0.739	0.497	0.759

**Table 2:** Results of the two-way ANOVA (*p*-values) for growth and photosynthetic performance in tomato plants nine weeks after transplanting with the factors ‘*Serendipita*’, ‘AMF,’ and ‘*Serendipita* × AMF’ interactions (*p* < 0.05, *n* = 15).

Factor	Growth		Performance of Photosystem II		
	Shoot Length	Shoot Dry Weight	Fo ^1^	Fm ^2^	Fv/Fm ^3^
*Serendipita*	0.214	0.365	0.137	0.949	0.191
AMF	0.079	0.536	0.809	0.197	**0.025**
*Serendipita* × AMF	0.121	0.063	**0.010 ^4^**	**0.045**	0.467

^1^ Fo—minimal & ^2^ Fm—maximal chlorophyll fluorescence yield; ^3^ Fv/Fm—maximum photochemical quantum yield of photosystem II. ^4^ Significant values (*p* < 0.05) are highlighted in bold.

**Table 3:** Mean values (±S.D.) of the above ground growth of tomato plants nine weeks after transplanting (*n* = 15).

Treatments	Growth	
	Shoot Length (cm)	Shoot DW (g)
Control	23.09 ± 1.32	1.16 ± 0.25
*S. indica*	24.48 ± 1.86	1.20 ± 0.15
*S. williamsii*	22.81 ± 2.38	1.22 ± 0.22
AMF	22.91 ± 1.79	1.21 ± 0.11
*S. indica* + AMF	22.69 ± 1.69	1.23 ±0.13
*S. williamsii* + AMF	22.76 ± 1.61	1.07 ± 0.15

**Table 4:** Results of the two-way ANOVA (*p*-values) for macronutrient concentrations in tomato shoots nine weeks after transplanting with the factors ‘*Serendipita*’, ‘AMF’, and ‘*Serendipita* × AMF’ interaction (*p* < 0.05, *n* = 4).

Factor	Macronutrients						
	P	Ca	Mg	K	C	N	CN
*Serendipita*	0.281	0.094	0.227	0.511	0.841	0.461	0.595
AMF	**0.002 ^1^**	**0.001**	0.144	0.782	0.475	0.985	0.894
*Serendipita* × AMF	0.085	**0.012**	0.136	0.061	**0.014**	**0.001**	**0.000**

^1^ Significant values (*p* < 0.05) are highlighted in bold.

**Table 5:** Results of the two-way ANOVA (*p*-values) for micronutrient concentration in the tomato shoots nine weeks after transplanting with the factors ‘*Serendipita*’, ‘AMF’, and ‘*Serendipita* × AMF’ interactions (*p* < 0.05, *n* = 4).

Treatments	Micronutrients		
	Mn	Fe	Zn
*Serendipita*	0.554	0.612	0.306
AMF	**0.000 ^1^**	**0.000**	0.720
*Serendipita* × AMF	0.086	0.345	0.176

^1^ Significant values (*p* < 0.05) are highlighted in bold.
